# Case Report: A case of cutaneous collagenous vasculopathy

**DOI:** 10.3389/fmed.2026.1899277

**Published:** 2026-09-03

**Authors:** Chenjian Tang, Liping Chen, Jiangxiu Yu, Mei Zhu

**Affiliations:** 1 Department of Acupuncture, Hospital of Chengdu University of Traditional Chinese Medicine, Chengdu, China; 2 Department of Geriatrics, People’s North Road Community Health Service Center (The Second People's Hospital of Jinniu District), Chengdu, China; 3 Department of Dermatology, Ba Zhong Hospital of Integrated Traditional Chinese and Western Medicine, Chengdu, China; 4 Department of Dermatology, Hospital of Chengdu University of Traditional Chinese Medicine, Chengdu, China

**Keywords:** allergic purpura, collagenous vasculopathy, cutaneous, misdiagnose, mistreatment

## Abstract

We report a case of a 62-year-old woman presenting with a 6-year history of purpuric patches and macules on the lower extremities, initially misdiagnosed as allergic purpura and treated with prednisone. Skin biopsy revealed dilated dermal vessels with perivascular collagen deposition, positive for collagen IV and diastase–periodic acid–Schiff (D-PAS) staining, consistent with cutaneous collagenous vasculopathy (CCV). Treatment included hydroxychloroquine, vitamin C, doxepin, and topical mucopolysaccharide polysulfate cream. Prednisone was tapered by one tablet every 3 days. After 4 weeks, the rash improved significantly with no new lesions. We would like to present a case of CCV.

## Introduction

Cutaneous collagenous vasculopathy (CCV) is a rare, acquired microangiopathy characterized by generalized telangiectasia with perivascular collagen deposition (1, 2). CCV often presents asymptomatically and can be misdiagnosed as other vascular disorders. The pathogenesis remains unclear, but it involves abnormalities in vascular wall integrity leading to collagen accumulation (3).

## Case report

A 62-year-old woman was admitted with a 6-year history of purpuric patches and macules on both lower extremities, worsening over 1 month. The lesions were non-blanchable, associated with pruritus, but without joint pain or abdominal discomfort. Past medical history included well-controlled hypertension treated with amlodipine. Initially, she was diagnosed with allergic purpura and treated with prednisone 30 mg daily for over 6 months without dose reduction. The patient exhibited only a minimal response to prednisone, with transient improvement in symptoms that did not lead to sustained disease control.

**Figure 1:**
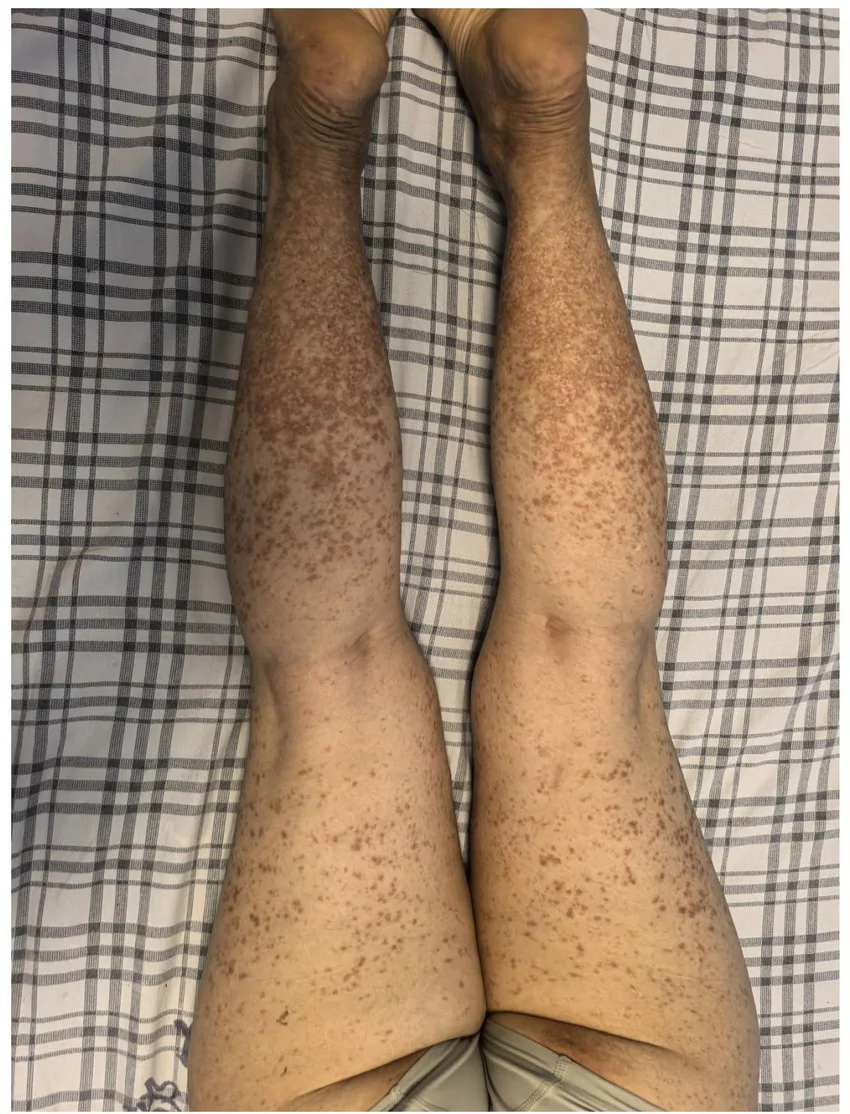
Two legs lying flat on a checkered fabric surface with numerous small, dark, reddish-brown spots distributed across the skin from thighs to feet.

**Figure 2:**
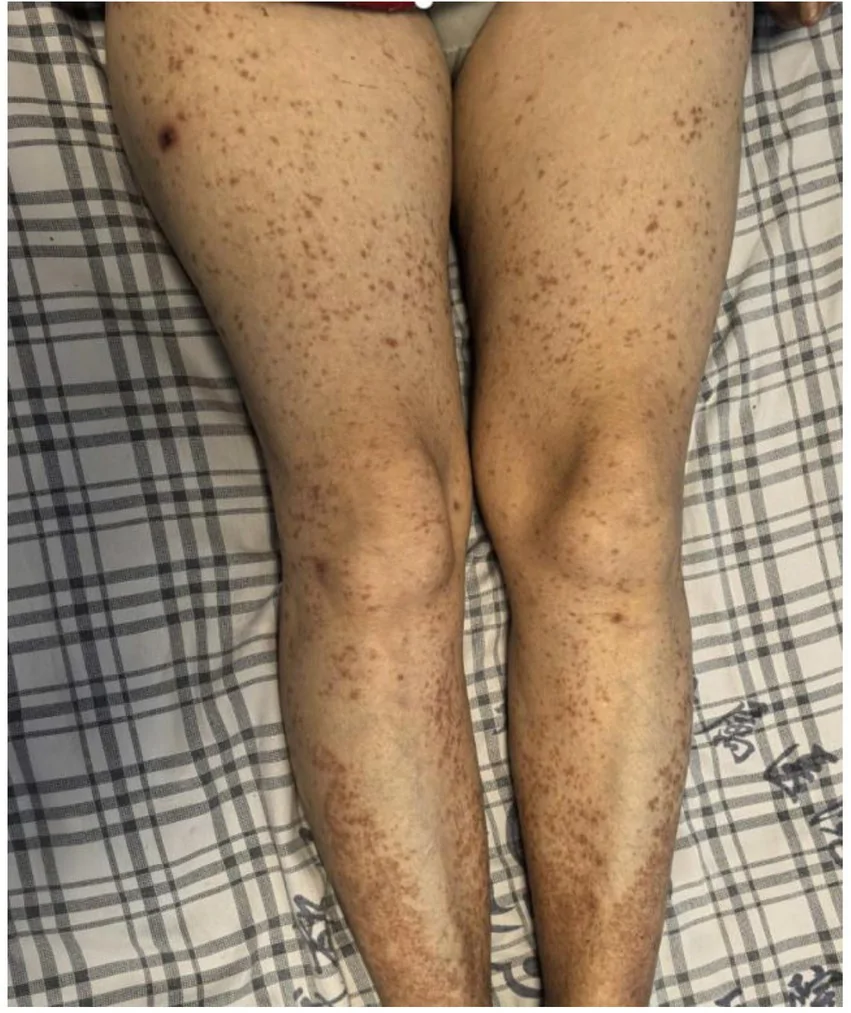
Two outstretched legs lying on a plaid patterned surface exhibit widespread dark brown spots and lesions concentrated on the thighs, knees, and shins.

Purpuric lesions were observed on the lower limbs (Figures 1, 2), with normal skin temperature and no signs of systemic involvement. Laboratory investigations, including complete blood count, coagulation tests, immune profiles (e.g., antinuclear antibody, anti-RNP antibody, anti-Sm antibody, anti-SSA antibody, anti-SSB antibody, anti-Ro-52 antibody, anti-Scl-70 antibody, anti-Jo-1 antibody, anti-centromere antibody, anti-nucleosome antibody, and anti-ribosomal P protein antibody), renal and hepatic functions, urinalysis, and infectious disease screening (hepatitis, human immunodeficiency virus (HIV), syphilis), were all within normal limits. A 24-h urine protein quantification and stool tests were unremarkable.

**Figure 3:**
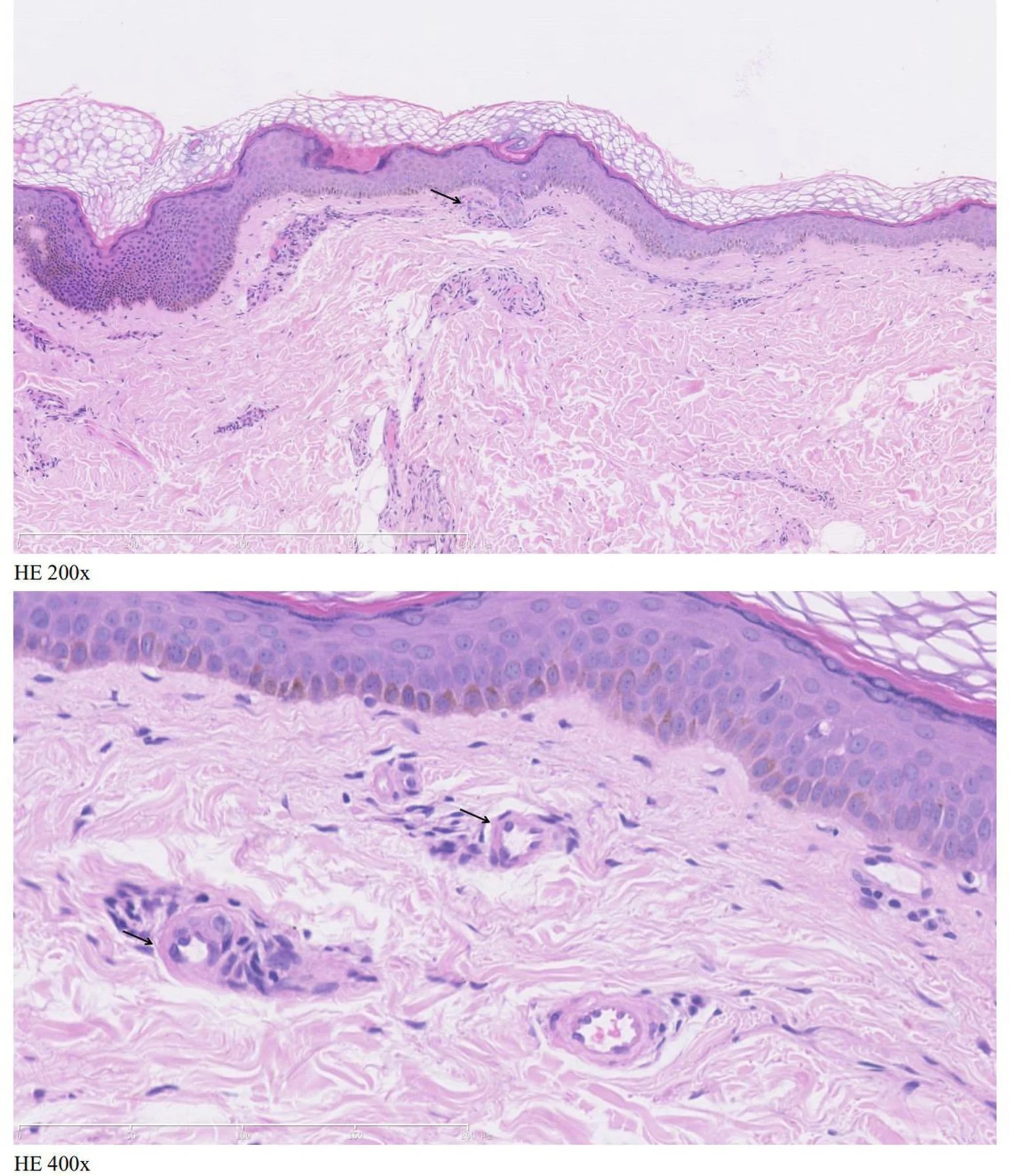
Histological sections of human skin stained with hematoxylin and eosin at two magnifications display the epidermis and dermis.

**Figure 4:**
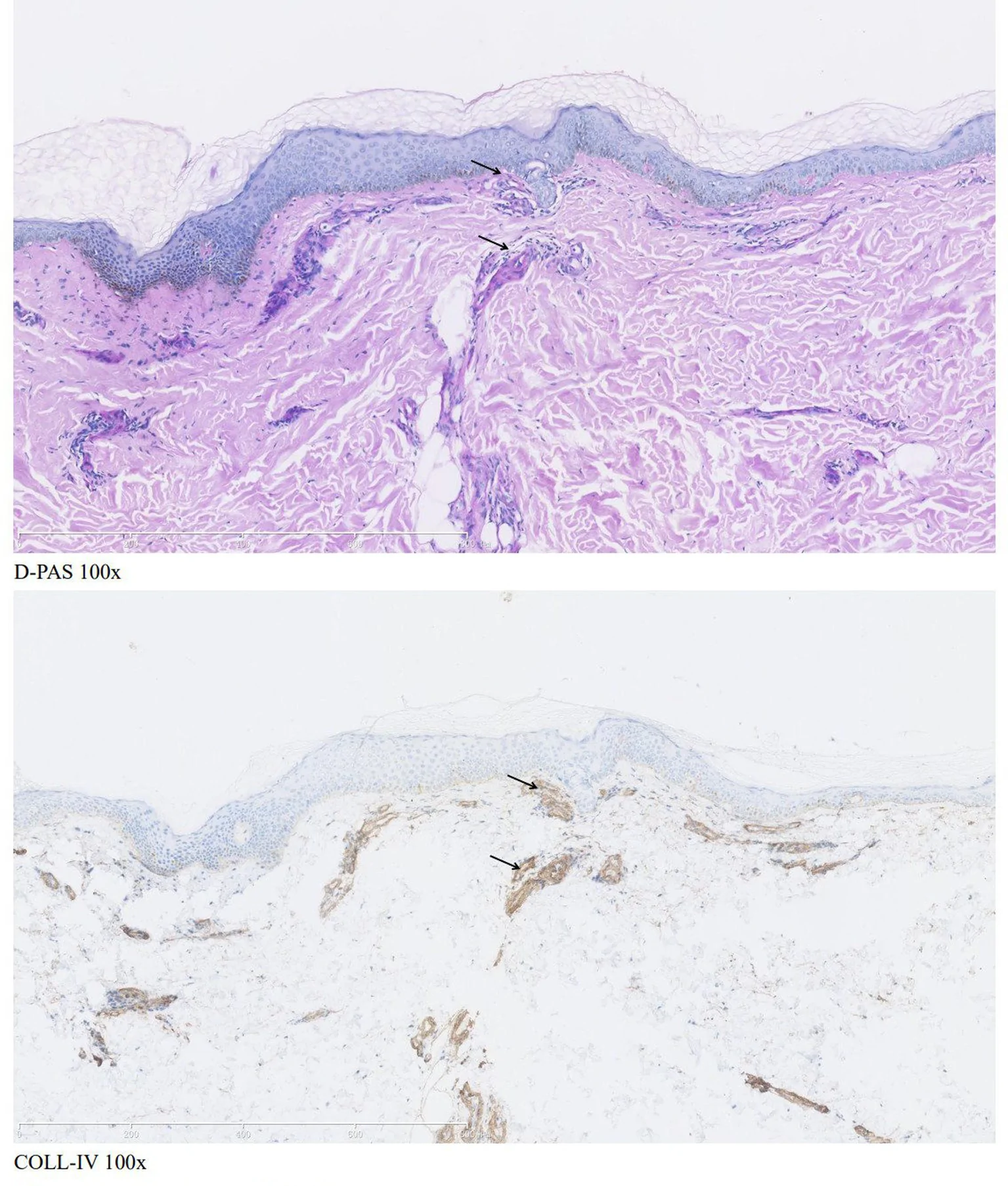
Microscope slide images of skin tissue stained by two different methods at one hundred times magnification, with black arrows indicating similar regions of interest beneath the epidermis for both D-PAS and COLL-IV stains.

Skin biopsy (Figure 3) of the right thigh showed normal epidermis with dilated small vessels in the superficial dermis and mild perivascular lymphocytic infiltration. Notably, there was no evidence of vasculitis, erythrocyte extravasation, or hemosiderin deposition. Giemsa staining indicated no significant mast cell increase, and iron stain was negative. Immunohistochemistry revealed collagen IV positivity around vessels (Figure 4), indicating perivascular thickening of hyaline, transparent material in the endothelium, and diastase–periodic acid–Schiff (D-PAS) staining highlighted thickened perivascular basement membrane-like deposits (Figure 4). Based on these findings, a diagnosis of CCV was established.

Treatment included hydroxychloroquine, vitamin C, doxepin, and topical mucopolysaccharide polysulfate cream. Prednisone was tapered by one tablet every 3 days. After four weeks, the rash improved significantly with no new lesions. Therefore, prednisone was discontinued. The patient remains under follow-up.

## Discussion

Cutaneous collagenous vasculopathy (CCV) is a rare, idiopathic microangiopathy primarily affecting postcapillary venules, first described by Salama and Rosenthal (1). This disease has only been reported in a few cases so far, and it mainly affects middle-aged and older adults (4, 5). The present case involves a 62-year-old woman with a 6-year history of purpuric patches on both lower extremities, initially misdiagnosed as allergic purpura and treated with prolonged high-dose prednisone. This diagnostic challenge is not uncommon, as CCV clinically mimics several telangiectatic disorders. The differential diagnosis includes generalized essential telangiectasia (GET), hereditary hemorrhagic telangiectasia (HHT), unilateral nevoid telangiectasia, pigmented purpuric dermatoses (PPD), and angioma serpiginosum. GET typically presents with more widespread telangiectasias involving mucous membranes and shows thin-walled dilated vessels on histopathology, without the characteristic thickened vessel walls seen in CCV (6). HHT is an autosomal dominant disorder associated with recurrent epistaxis and visceral involvement, with a positive family history (2). PPD, while sharing purpuric features, demonstrates erythrocyte extravasation and hemosiderin deposition on histology, which were notably absent in our patient (7). The absence of vasculitis, erythrocyte extravasation, and iron deposition further ruled out other purpuric conditions.

The pathogenesis of CCV remains incompletely understood. Proposed mechanisms include repetitive endothelial cell injury leading to microthrombus formation, compensatory endothelial proliferation, and subsequent perivascular fibrosis (8). The reduction or absence of pericytes around affected vessels, along with loss of contractile filaments within residual pericytes, may contribute to vascular dilation and impaired vasoconstriction (1, 9). Additionally, abnormal perivascular collagen deposition may weaken structural support, further promoting ectasia. Some studies have suggested associations with systemic conditions such as diabetes mellitus, hypertension, autoimmune diseases, and medication use (e.g., calcium channel blockers, interferons, hydroxyurea) (10, 11), though causal relationships remain unestablished.

Treatment options for CCV are limited, with no established consensus or guidelines. Pulsed-dye laser (PDL) and neodymium-doped yttrium aluminum garnet (Nd:YAG) laser have shown efficacy in improving cosmetic appearance by targeting dilated vessels (12–15). However, recurrences may occur, and treatment response varies. Pharmacological interventions remain anecdotal. These therapeutic measures were selected in light of our clinical consideration of a collagen vascular disease. Vitamin C was administered to reduce vascular permeability. Given that the patient experienced pruritus, doxepin was prescribed for its antipruritic effects. Additionally, topical mucopolysaccharide polysulfate was employed for its antithrombotic and anti-inflammatory actions. While hydroxychloroquine has not been systematically studied in CCV, its anti-inflammatory and immunomodulatory effects may theoretically benefit patients with suspected immune-mediated microangiopathy. Vitamin C and doxepin may reduce vascular permeability. Topical application of mucopolysaccharide polysulfate can inhibit enzymes involved in catabolism and affect the prostaglandin and complement systems, thereby preventing the progression of local inflammation.

In conclusion, CCV should be considered in the differential diagnosis of acquired, progressive, asymptomatic telangiectasias or purpuric macules. Misdiagnosis can lead to unnecessary immunosuppressive therapy, as illustrated by our patient’s prolonged prednisone use.

## Data Availability

The original contributions presented in the study are included in the article/supplementary material, further inquiries can be directed to the corresponding author.
